# Exome sequencing identifies novel dysferlin mutation in a family with pauci-symptomatic heterozygous carriers

**DOI:** 10.1186/s12881-018-0613-x

**Published:** 2018-06-07

**Authors:** Mahjoubeh Jalali-Sefid-Dashti, Melissa Nel, Jeannine M. Heckmann, Junaid Gamieldien

**Affiliations:** 1 South African Medical Research Council Bioinformatics Unit, South African National Bioinformatics Institute, University of the Western Cape, Bellville, 7535 South Africa; 2 Division of Neurology, Department of Medicine, University of Cape Town, Observatory, 7925 South Africa; 3 E8-74, Neurology, New Groote Schuur Hospital Observatory, Cape Town, 7925 South Africa; 4 South African National Bioinformatics Institute, University of the Western Cape, Private Bag X17, Bellville, 7535 South Africa

**Keywords:** Exome, Dysferlinopathy, Myalgia, Cramps, Pauci-symptomatic carriers

## Abstract

**Background:**

We investigated a South African family of admixed ancestry in which the first generation (G1) developed insidious progressive distal to proximal weakness in their twenties, while their offspring (G2) experienced severe unexpected symptoms of myalgia and cramps since adolescence. Our aim was to identify deleterious mutations that segregate with the affected individuals in this family.

**Methods:**

Exome sequencing was performed on five cases, which included three affected G1 siblings and two pauci-symptomatic G2 offspring. As controls we included an unaffected G1 sibling and a spouse of one of the G1 affected individuals. Homozygous or potentially compound heterozygous variants that were predicted to be functional and segregated with the affected G1 siblings, were further evaluated. Additionally, we considered variants in all genes segregating exclusively with the affected (G1) and pauci-symptomatic (G2) individuals to address the possibility of a pseudo-autosomal dominant inheritance pattern in this family.

**Results:**

All affected G1 individuals were homozygous for a novel truncating p.Tyr1433Ter *DYSF* (dysferlin) mutation, with their asymptomatic sibling and both pauci-symptomatic G2 offspring carrying only a single mutant allele. Sanger sequencing confirmed segregation of the variant. No additional potentially contributing variant was found in the *DYSF* or any other relevant gene in the pauci-symptomatic carriers.

**Conclusion:**

Our finding of a truncating dysferlin mutation confirmed dysferlinopathy in this family and we propose that the single mutant allele is the primary contributor to the neuromuscular symptoms seen in the second-generation pauci-symptomatic carriers.

## Background

Dysferlinopathies are a group of autosomal recessive muscular dystrophies caused by mutations in the dysferlin gene, *DYSF* [1, 2], and is typified by markedly reduced or absent dysferlin protein on immunohistochemical staining in muscle [3]. It most frequently presents as a distal myopathy affecting first the posterior distal leg compartment (Miyoshi muscle dystrophy), limb-girdle muscle dystrophy (LMGD2B) and a combination of the aforementioned, “proximodistal” myopathy [4, 5]. Clinical manifestations can be significantly varied even between individuals in a family bearing the same mutation, which indicates a possible role for genetic modifiers [6–8].

A definitive diagnosis of dysferlinopathy can only be made when pathogenic mutations are identified in the large (>233Kbp, 58 exons, >6Kbp coding sequence) dysferlin (*DYSF*) gene [9], which lacks mutation hotspots. It was recently demonstrated that next generation sequencing (NGS) targeting the coding regions of the *DYSF* gene enables efficient and accurate genetic diagnosis of dysferlinopathy [10]. Whole exome sequencing (WES), however, may provide additional advantages as an unbiased diagnostic strategy in families requesting a definitive diagnosis and genetic counselling [11], particularly in atypical disease presentations and/or when unexpected phenotypes are suspected.

We present an exome sequencing study in a non-consanguineous family actively seeking a diagnosis for the neuromuscular symptoms experienced by themselves and their children over a period of 25 years. The first generation (G1) presented with a predominant distal ‘posterior calf’ myopathy starting in early adulthood. This was suggestive of classical autosomal recessive Miyoshi myopathy caused by compound heterozygous or homozygous dysferlin gene mutation(s), since neither of their parents had been diagnosed with muscular dystrophy. However, at least five of the second generation (G2) offspring had been attending neurological services with mainly exercise-induced muscle cramps over a number of years starting in adolescence.

## Methods

### Patients and clinical evaluations

The study and subject consent forms were approved by the University of Cape Town Health Sciences faculty human ethics research committee (REF 552/2013) and the study carried out in accordance with the approved guidelines and regulations. We studied two generations of a South African family of mixed genetic ancestry that likely includes ancestors from Africa, Europe as well as Madagascar and Java as previously described [12]. Family members of G1 and G2 had been attending the adult and paediatric neurology clinics attached to the University of Cape Town for 25 years. After signed informed consent, blood was obtained for WES from G1 individuals, three affected (I-2, I-4 and I-5), one unaffected sibling (I-1) and one unrelated unaffected family member (I-3) as controls, and two members from G2 with neuromuscular symptoms and areflexia, II-2 and II-5. Structured folder reviews were performed to obtain previous examination details, laboratory data including those of muscle biopsies and clinical electrophysiological studies.

### Whole exome capture, sequencing and variant calling

Paired-end exome sequencing was performed at 50× coverage by CLIA accredited Otogenetics Corporation, Norcross, GA, USA using the Agilent SureSelect Human V5 + UTR capture kit and the Illumina HiSeq2000/2500 platform. After quality control, reads for each patient were aligned to the hg19 human reference genome using NOVOALIGN [13], PCR duplicates were removed using Picard [14], followed by indel realignment and base quality score recalibration, variant calling and quality evaluation using the Genome Analysis ToolKit [15] version 3.6 to produce a high-confidence set of variants for each sample.

### Identification of likely function-impacting candidate variants

Variants were annotated using ANNOVAR [16] and were filtered based on both autosomal recessive and dominant inheritance models. For the recessive model, we identified variants that were homozygous in the three affected G1 siblings, wild-type in their unaffected sibling, and wild-type or heterozygous in the unrelated unaffected family member and the G2 offspring. For the autosomal dominant model, we identified variants that were heterozygous in all the affected/pauci-symptomatic family members, and where both related and unrelated controls were wild-type. For initial filtering, variants with a minor allele frequency > 1% in public databases, namely the 1000 Genomes Project [17], the NHLBI-ESP 6500 Exome Sequencing Project [18] and the Exome Aggregation Consortium [19] databases were filtered out, as were those present in the currently unreleased Southern African Human Genome Program [20] variant dataset. Nonsense, frameshift and splicing variants were automatically selected as preliminary candidates, while missense variants were further evaluated if they passed the recommended deleteriousness score thresholds for any one of FATHMM [21] or the MetaSVM and MetaLR ensemble prediction methods [22].

## Results

### Clinical findings

**Fig. 1:**
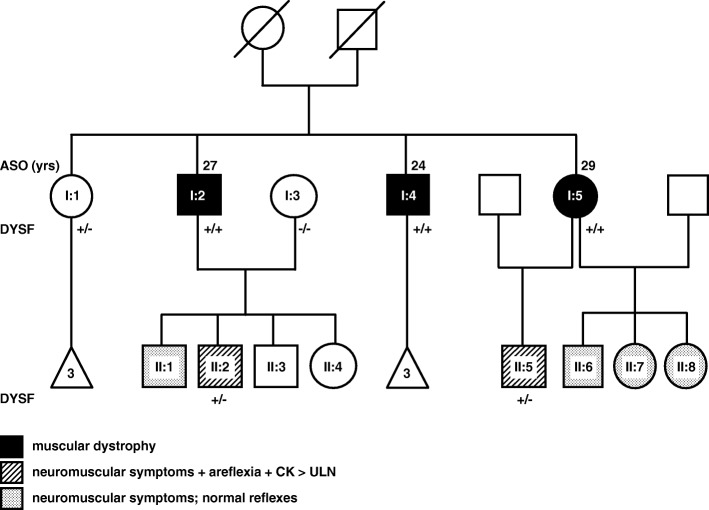
Family pedigree and validated genotypes for the novel DYSF stop gain variants. Circles represent females and squares represent males. White figures (excluding triangles) symbolize unaffected members; solid symbolize disease cases, striped represent cases with neuromuscular symptoms, areflexia and elevated creatine kinase (CK) levels above the upper limit of normal (ULN); and speckled symbolize cases with neuromuscular symptoms but normal deep tendon reflexes. Individuals denoted by triangles were of uncertain clinical status and “/” indicate deceased individuals. ASO refers to age at symptom onset in years (yrs). Generation 1 (G1) refers to I:1 to I:5 and G2 refers to II:1 to II:8. DYSF refers to the dysferlin gene. ‘+’ refers to the presence of the DYSF mutation, ‘-‘to wild-type DYSF, +/− refers to heterozygous individuals and +/+ to homozygous individuals

Three affected individuals from G1 had developed progressive muscle weakness in their twenties, two of whom also had prominent exercise-induced myalgia, and five individuals from the second generation developed neuromuscular symptoms in adolescence (Fig. 1). Two individuals from G1 (I-4 and I-5) presented with the distal Miyoshi muscle dystrophy phenotype and one (I-2) with a “proximodistal” phenotype. Two cases from G2 had areflexia and marginally raised CK (II-2 and II-5), and three (II-1, II-6, II-7 and II-8) had normal reflexes. Individuals I-1, II-3, and II-4 had no neuromuscular symptoms or signs. The offspring of 1–1 were asymptomatic adults, and those of I-4 had not reached adolescence and were not examined.

The index case (I-4), first examined at age 26, complained of two years of progressive thinning of the distal legs and quadriceps, an inability to stand on his toes, and myalgia in his legs aggravated by exercise. Later, he noticed weakness in the hands. The clinical presentation was a distal, posterior compartment muscular dystrophy with markedly raised creatine kinase (CK) levels (> 47× upper limit of normal (ULN)). However, the tendon reflexes were either absent (legs) or reduced (arms), and four years later all the reflexes were absent. Electrophysiology showed normal nerve conductions, and myopathic features on needle electromyography (EMG). Electrocardiography was normal. A deltoid muscle biopsy confirmed a dystrophic process without inflammatory infiltrates; no special staining was available. At that time, his two asymptomatic sisters (one was Case I-5, aged 21, see below) and mother were noted to have normal CK levels (CK ≈ 61 IU/L; *N* = 26–140 IU/L), although his father, aged 51, complained of muscle cramps and had a slightly raised CK level (249 IU/L) which was 1.5× above the upper level of normal expected for age and sex. Approximately 15 years after symptom onset, I-4 required bilateral crutches to mobilize, and 10 years later became wheelchair bound. The pattern of weakness had progressed to severe limb girdle and distal weakness. Although sensation was previously recorded as intact, the last examination at age 49 showed evidence of a mild sensory stocking neuropathy.

Case I-2 presented at age 29 with a history of progressive leg weakness since mid-twenties, noting difficulty climbing stairs, getting up from chairs and exercise-induced muscle cramps, especially in his calves. His examination showed wasting of the biceps and distal legs, mild proximo-distal posterior leg weakness, and reduced/absent reflexes. Later he exhibited a waddling gait. The CK level was 20× ULN. A muscle biopsy at age 40 showed features of muscle dystrophy and immunohistochemistry showed absence of dysferlin in the presence of positive merosin, emerin, caveolin, dystrophin and sarcoglycan staining. Fifteen years later he required crutches to mobilize.

Case I-5 presented at age 34 with increasing difficulty in walking, climbing stairs, rising from a seated position, and general muscle fatigue since her late twenties. The arms showed tapering distally and the posterior compartment of the legs, marked wasting. Her tendon reflexes were globally depressed and there was marked weakness of the posterior compartment leg muscles. Her CK level was > 30× ULN. A muscle biopsy of the left biceps showed similar results to her brother except that dysferlin staining was initially present and dystrophin was absent, but the positive control (spectrin) showed partial staining. Her disability increased substantially and at age 45 years she was largely confined to a wheelchair. A repeat biopsy of the right biceps showed absent dysferlin staining.

II-2 was examined at age 18 years. He had normal early motor development but was noted to fall more than usual as a child whilst running. Muscle cramps and stiffness, especially with physical activity, was noted during early adolescence. At age 15 he had increasing difficulty with riding his bicycle, climbing stairs as well as gait instability and stopped playing sport. He fell frequently. The neurological examination showed mild wasting of the biceps despite well-developed muscles elsewhere. His tendon jerks were absent. Power testing was normal, but he had a mild waddling gait. Sensation and coordination testing were normal. Clinical electrophysiology was refused. The CK level was at the upper the limit of normal (ULN) for his age (218 IU/L).

II-5 had experienced muscle pain, and episodes of cramp and stiffness lasting several hours to 1–2 days since the age of 12–13 years. These symptoms were, and still are, aggravated by physical activity. Since his early twenties he has also noticed increasing clumsy ankles and occasional give-in weakness of the legs. Examination at age 25 showed floppy ankles but no obvious wasting in his hands or feet. His tendon reflexes were globally absent and he had mild weakness of toe flexors but not of the plantar flexors. The remaining motor, sensory (all modalities) and coordination systems were normal. The CK level was slightly elevated (193 IU/L; 1.1× ULN for age and sex). Nerve conduction studies were normal. EMG of the medial head of gastrocnemius showed no spontaneous activity and normal motor units.

Cases II-1, II-6, II-7 and II-8 had been experiencing neuromuscular symptoms since the ages of 12 to 13 years, mainly myalgia, stiffness and/or muscle cramps. These symptoms occurred either in the hands or legs and were frequently provoked by mild physical activity including writing with a pen or walking upstairs, respectively.

### Exome sequencing and filtering for candidate deleterious variants

**Fig. 2:**
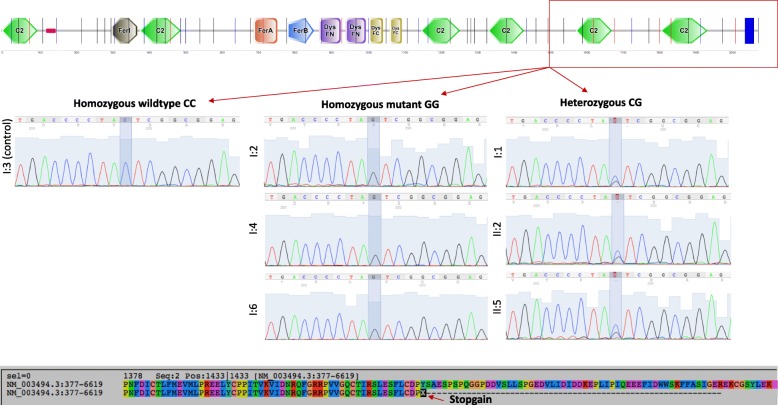
Protein level consequence of the identified novel stop-gain dysferlin gene (*DYSF*) mutation. The diagram illustrates the loss of two C2 domains in *DYSF* gene due to the identified novel stop-gain. The chromatogram illustrates the Sanger sequencing validation for mutant and wild-type alleles in the affected and unaffected family members. The protein alignment illustrates the nonsense mutation and protein truncation

WES was performed on I-2, I-4 and I-5 from G1, one unaffected sibling (I-1) and one unrelated unaffected family member (I-3) as controls, and on two members from G2 with neuromuscular symptoms and areflexia, II-2 and II-5. Approximately 50,000 variants were identified for each individual. Six variants segregated with all three symptomatic G1 individuals using the recessive model, of which three were in exonic regions, and one each in a 3′-untranslated region (UTR), intronic and intergenic regions, respectively. A novel nonsense *C > G* mutation located at position 4299 in exon 39 (NCBI RefSeq: NM_003494) of the dysferlin gene (*DYSF*) was identified as responsible for the family’s muscular dystrophy after filtering out common variants and those not predicted to impact protein function. Sanger sequencing confirmed the mutation to be homozygous in affected individuals of G1, and carrier status in I-1, II-2 and II-5. The p.Tyr1433Ter (NP_003485.1) mutation results in the loss of the sixth (C2E) and seventh (C2F) domains of the dysferlin protein (Fig. 2).

No other potentially deleterious dysferlin variant was found in the exomes of the two pauci-symptomatic G2 *DYSF*-mutant carriers. As they also presented with neuromuscular symptoms and areflexia in absence of distinct myopathy, we further filtered for rare or novel variants predicted to impact protein function that segregated exclusively with symptomatic individuals in both G1 and G2. Only two novel heterozygous missense mutations were identified in the *LRP2* (low density lipoprotein-related protein 2) and *GXYLT1* (glucoside xylosyltransferase 1) genes, which have no known roles in neuromuscular disease.

## Discussion

The G1 symptomatic individuals developed mainly distal posterior compartment myopathy in their legs during their early twenties with extremely high CK levels at the time of diagnosis. The first generation’s symptoms were indicative of Miyoshi myopathy/dysferlinopathy, since neither of their parents was diagnosed with myopathy even after age 60. Using WES, we confirmed autosomal recessive dysferlinopathy in this South African family, caused by a novel truncating p.Tyr1433Ter mutation in the *DYSF* gene, which accounts for the muscle dystrophy in the first generation. An asymptomatic G1 sibling also carried one copy of the *DYSF* mutation.

Several of their offspring experienced neuromuscular symptoms starting in adolescence to the extent that they had been followed at the pediatric neurology service for several years. The current study found that all the G2 cases with neuromuscular symptoms have only one copy of the novel nonsense *DYSF* mutation and no other potentially disease-causing variants in the gene. Although we postulated that co-occurrence of another mutation in a second gene could potentially be causing or contributing to the neuromuscular symptoms observed in G2, we only found two novel heterozygous missense mutations in the *LRP2* and *GXYLT1* genes, neither of which are reasonable candidates based on their known cellular functions and disease associations. We therefore suggest that the single mutated *DYSF* allele could be responsible for the less severe neuromuscular symptoms in G2. Interestingly, of the two inferred heterozygous deceased parents, the father apparently also complained of neuromuscular symptoms (see Clinical Findings). The observation of a single mutant allele in both unaffected and pauci-symptomatic individuals suggests that variable penetrance, and/or other unidentified modifier allele(s), may underlie the manifestation of neuromuscular symptoms.

Fanin et al. [23] suggested in 2006 that carriers of *DYSF* mutations could be at risk of developing a milder phenotype and a number of reports have since contributed evidence to support this hypothesis. Two unrelated cases of *DYSF* mutation carriers presenting in middle age with muscle weakness, elevated creatine kinase, abnormal muscle MRI and reduced levels of muscle dysferlin, have been reported [24]. Another case of a bent spine syndrome/camptocormia, presenting in the seventh decade, appears to be an unusual presentation of pauci-symptomatic dysferlinopathy based on a heterozygous dysferlin mutation [25]. Another study was able to support a diagnosis of primary dysferlinopathy in symptomatic carriers with findings of abnormal dysferlin gene expression in skeletal muscle and monocytes [26]. Of note is that the two patients studied were unrelated but carried the same mutated allele, which suggests that certain mutations may have a higher likelihood than others of producing symptoms in carriers.

## Conclusion

We confirm dysferlinopathy in this family due to a novel truncating p.Tyr1433Ter *DYSF* mutation. Our report highlights the importance of considering variable penetrance of heterozygous dysferlin mutations in the context of pauci-symptomatic younger offspring.

## Abbreviations

ASO: Age of symptom onset
CK: Creatine kinase
CLIA: Clinical Laboratory Improvement Amendments
*DYSF*: Dysferlin
EMG: Electromyography
G1: First generation
G2: Second generation
*GXYLT1*: Glucoside xylosyltransferase 1
IUL: International Units Per Litre
LMGD2B: Limb-girdle muscular dystrophy
*LRP2*: Low density lipoprotein-related protein 2
MRI: Magnetic resonance imaging
NGS: Next generation sequencing
PCR: Polymerase chain reaction
ULN: Upper limit of normal
UTR: Untranslated region
WES: Whole exome sequencing

## References

[1] Bashir R, Britton S, Strachan T, Keers S, Vafiadaki E, Lako M (1998). A gene related to Caenorhabditis elegans spermatogenesis factor fer-1 is mutated in limb-girdle muscular dystrophy type 2B. Nat Genet.

[2] Liu J, Aoki M, Illa I, Wu C, Fardeau M, Angelini C (1998). Dysferlin, a novel skeletal muscle gene, is mutated in Miyoshi myopathy and limb girdle muscular dystrophy. Nat Genet.

[3] Klinge L, Aboumousa A, Eagle M, Hudson J, Sarkozy A, Vita G (2010). New aspects on patients affected by dysferlin deficient muscular dystrophy. J Neurol Neurosurg Psychiatry.

[4] Laval SH, Bushby KMD (2004). Limb-girdle muscular dystrophies--from genetics to molecular pathology. Neuropathol Appl Neurobiol.

[5] Nguyen K, Bassez G, Krahn M, Bernard R, Laforêt P, Labelle V (2007). Phenotypic study in 40 patients with dysferlin gene mutations: high frequency of atypical phenotypes. Arch Neurol.

[6] Weiler T, Bashir R, Anderson LV, Davison K, Moss JA, Britton S (1999). Identical mutation in patients with limb girdle muscular dystrophy type 2B or Miyoshi myopathy suggests a role for modifier gene(s). Hum Mol Genet.

[7] Illarioshkin SN, Ivanova-Smolenskaya IA, Greenberg CR, Nylen E, Sukhorukov VS, Poleshchuk VV (2000). Identical dysferlin mutation in limb-girdle muscular dystrophy type 2B and distal myopathy. Neurology.

[8] Ueyama H, Kumamoto T, Nagao S, Masuda T, Horinouchi H, Fujimoto S (2001). A new dysferlin gene mutation in two Japanese families with limb-girdle muscular dystrophy 2B and Miyoshi myopathy. Neuromuscul Disord.

[9] Tagawa K, Ogawa M, Kawabe K, Yamanaka G, Matsumura T, Goto K (2003). Protein and gene analyses of dysferlinopathy in a large group of Japanese muscular dystrophy patients. J Neurol Sci.

[10] Shin HY, Jang H, Han JH, Park HJ, Lee JH, Kim SW (2015). Targeted next-generation sequencing for the genetic diagnosis of dysferlinopathy. Neuromuscul Disord.

[11] Rehm HL (2013). Disease-targeted sequencing: a cornerstone in the clinic. Nat Rev Genet.

[12] Heckmann JM, Owen EP, Little F (2007). Myasthenia gravis in south Africans: racial differences in clinical manifestations. Neuromuscul Disord.

[13] Hansen NF (2016). Variant calling from next generation sequence data. Methods Mol Biol.

[14] BroadInstitute. Picard Tools - By Broad Institute [Internet]. 2016 [cited 2016 Jun 9]. Available from: http://broadinstitute.github.io/picard/

[15] McKenna A, Hanna M, Banks E, Sivachenko A, Cibulskis K, Kernytsky A (2010). The genome analysis Toolkit: a MapReduce framework for analyzing next-generation DNA sequencing data. Genome Res.

[16] Wang K, Li M, Hakonarson H (2010). ANNOVAR: functional annotation of genetic variants from high-throughput sequencing data. Nucleic Acids Res.

[17] 1000 Genomes Project Consortium {fname}, Abecasis GR, Auton A, Brooks LD, MA DP, Durbin RM, et al. An integrated map of genetic variation from 1,092 human genomes. Nature. 2012:491, 56–65. 10.1038/nature11632 PMC3498066 23128226

[18] Fu W, O’Connor TD, Jun G, Kang HM, Abecasis G, Leal SM (2013). Analysis of 6,515 exomes reveals the recent origin of most human protein-coding variants. Nature.

[19] Lek M, Karczewski KJ, Minikel EV, Samocha KE, Banks E, Fennell T (2016). Analysis of protein-coding genetic variation in 60,706 humans. Nature Nature Publishing Group.

[20] Pepper MS (2011). Launch of the southern African human genome Programme. South African Med J.

[21] Shihab HA, Rogers MF, Gough J, Mort M, Cooper DN, Day INM (2015). An integrative approach to predicting the functional effects of non-coding and coding sequence variation. Bioinformatics.

[22] Dong C, Wei P, Jian X, Gibbs R, Boerwinkle E, Wang K (2015). Comparison and integration of deleteriousness prediction methods for nonsynonymous SNVs in whole exome sequencing studies. Hum Mol Genet.

[23] Fanin M, Nascimbeni AC, Angelini C (2006). Muscle protein analysis in the detection of heterozygotes for recessive limb girdle muscular dystrophy type 2B and 2E. Neuromuscul Disord.

[24] Illa I, De Luna N, Dominguez-Perles R, Rojas-Garcia R, Paradas C, Palmer J (2007). Symptomatic dysferlin gene mutation carriers: characterization of two cases. Neurology.

[25] Gáti I, Danielsson O, Gunnarsson C, Vrethem M, Häggqvist B, Fredriksson B-A (2012). Bent spine syndrome: a phenotype of dysferlinopathy or a symptomatic DYSF gene mutation carrier. Eur Neurol.

[26] Meznaric M, Gonzalez-Quereda L, Gallardo E, de Luna N, Gallano P, Fanin M (2011). Abnormal expression of dysferlin in skeletal muscle and monocytes supports primary dysferlinopathy in patients with one mutated allele. Eur J Neurol.

